# Glucose deprivation in tuberous sclerosis complex-related tumors

**DOI:** 10.1186/2045-3701-1-34

**Published:** 2011-10-21

**Authors:** Xiuyun Jiang, Heidi L Kenerson, Raymond S Yeung

**Affiliations:** 1 Department of Surgery, University of Washington, Seattle, WA 98195, USA

**Keywords:** mTOR, 2-deoxyglucose, glycolysis, metabolism, rapamycin, ketone bodies, fatty acids

## Abstract

**Background:**

Cancer cells possess unique metabolic phenotypes that are determined by their underlying oncogenic pathways. Activation of the PI3K/Akt/mTOR signaling cascade promotes glycolysis and leads to glucose-dependence in tumors. In particular, cells with constitutive mTORC1 activity secondary to the loss of TSC1/TSC2 function are prone to undergo apoptosis upon glucose withdrawal *in vitro*, but this concept has not been tested *in vivo*. This study examines the effects of restricting glucose metabolism by pharmacologic and dietary means in a tuberous sclerosis complex (TSC) tumor xenograft model.

**Results:**

Tumor-bearing mice were randomly assigned to receive unrestricted carbohydrate-free ("Carb-free") or Western-style diet in the absence or presence of 2-deoxyglucose (2-DG) in one of four treatment groups. After 14 weeks, tumor sizes were significantly different among the four treatment groups with those receiving 2-DG having the smallest tumors. Unexpectedly, the "Carb-free" diet was associated with the largest tumors but they remained responsive to 2-DG. PET imaging showed significant treatment-related changes in tumor ^18^fluorodeoxyglucose-uptake but the standard uptake values did not correlate with tumor size. Alternative energy substrates such as ketone bodies and monounsaturated oleic acid supported the growth of the *Tsc2*-/- cells *in vitro*, whereas saturated palmitic acid was toxic. Correspondingly, tumors in the high-fat, "Carb-free" group showed greater necrosis and liquefaction that contributed to their larger sizes. In contrast, 2-DG treatment significantly reduced tumor cell proliferation, increased metabolic stress (i.e., ketonemia) and AMPK activity, whereas rapamycin primarily reduced cell size.

**Conclusions:**

Our data support the concept of glycolytic inhibition as a therapeutic approach in TSC whereas dietary withdrawal of carbohydrates was not effective.

## Background

Tuberous sclerosis is an autosomal dominant disorder characterized by multiple benign hamartomas and neoplasms caused by the disruption of a pair of tumor suppressor genes, TSC1 and TSC2, which encode for hamartin and tuberin, respectively [1]. Mutations and epigenetic silencing of these genes have been reported in sporadic human cancers including epithelial tumors of the bladder, liver, and oral cavity as well as PEComas [2-6]. The TSC1 and TSC2 proteins negatively regulate mTOR Complex 1 (mTORC1) by inhibiting Rheb activity [7]. Consequently, mTORC1 is constitutively activated in cells lacking TSC1 or TSC2. These findings led to the use of rapamycin and its analogs in the treatment of TSC and related disorders [8-11]. The effect of rapamycin is cytostatic, and tumors re-grow upon cessation of treatment. Long-term rapamycin can cause significant side effects, thus alternative approaches are being investigated.

Oncogenic pathways such as PI3K/Akt and Myc promote aerobic glycolysis and glutaminolysis, respectively, to provide adequate supplies of ATP and substrates for macromolecular synthesis [12-15]. The dependence of tumor growth on these metabolic events provides a basis for metabolic intervention as a strategy for controlling tumors [16]. In this study, we examined the *in vivo *role of glucose deprivation in TSC-related tumors. Cells lacking hamartin or tuberin are prone to undergo apoptosis under low-glucose condition [17,18]. mTORC1 enhances aerobic glycolysis and lactate production via up-regulation of HIF1α [19]. TSC1/TSC2-null cells also exhibit impaired insulin-stimulated glucose uptake secondary to Glut4 mislocalization [20]. Pathology associated with TSC such as angiomyolipoma and lymphangioleiomyomatosis display low FDG uptake on PET imaging despite increased glycolytic activity [20,21]. The imbalance between energy supply and demand presents a rationale for targeting glucose metabolism to control mTORC1-mediated tumorigenesis.

Two common approaches to limit glucose metabolism in tumors include utilization of glycolytic inhibitors and dietary restriction. Compounds such as 2-deoxyglucose (2-DG) reduce cellular ATP levels and promote apoptosis especially in cells with mitochondrial respiration defects or under hypoxic condition [22,23]. Early clinical experience suggests that 2-DG is safe up to a dose of 250 mg/kg [24], but efficacy has not been well documented. Dietary restriction of glucose/carbohydrate (e.g., Atkins-type diets) leads to relative hypoglycemia, hypoinsulinemia and ketonemia in humans [25]. Ketogenesis is an ancient pathway of metabolic adaptation exploited when an organism experiences protracted energy stress [26]. Critical tissues such as the brain and kidney can efficiently metabolize ketone bodies, but it is unclear if tumor cells exhibit such adaptation.

Here, we studied the effects of 2-DG and a carbohydrate-free ("Carb-free") diet on the growth of mTORC1-dependent tumors using a *Tsc2-/- *xenograft model. The inhibition of glycolysis using 2-DG resulted in reduced cell proliferation and suppressed tumor growth, thus confirming the sensitivity of TSC-related tumors to metabolic intervention. On the other hand, the "Carb-free" diet failed to promote ketogenesis and led to increased tumor size despite reduction in body weights. Our study highlights the differential effects of glycolytic inhibition and dietary glucose deprivation in modulating tumor metabolism and growth.

## Results

### Sensitivity of Tsc2-/- cells to 2-DG

**Figure 1:**
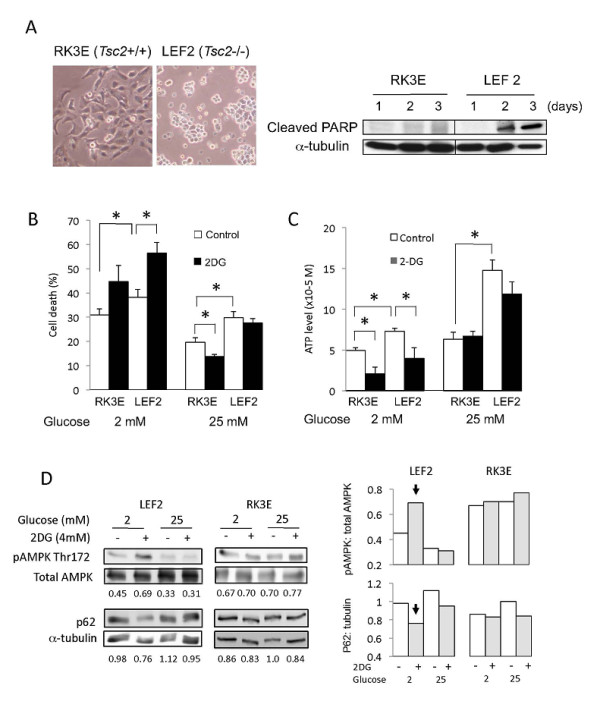
**Effects of 2-DG on LEF2 cells**. A) Wild-type RK3E and *Tsc2-/- *LEF2 cells were grown in 10%FBS DMEM with 2 mM glucose. Left: Phase contrast photomicrographs of cells after 2 days. Magnification 10×. Right: Immunoblot of cell lysates showing expression of cleaved PARP and α-tubulin at indicated times. Effects of glucose concentration and 2-DG (4 mM) for 6 hours on B) cell viability as determined by Annexin V/PI flow cytometric analyses, C) cellular ATP levels (Luminescence assay, PerkinElmer), error bars represent SEM. *, p < 0.05, and D) AMPK phosphorylation and p62 expression by Western blotting. The relative expression of these proteins were analyzed by densitometry, and the numbers under the blots indicate the ratio of band intensities (Image J) between p-AMPK:total AMPK and p62:α-tubulin. The densitometry results are also shown in graphical form on the right. Arrows indicate the most significant changes.

Our *Tsc2*-null tumor xenograft model utilizes tumorigenic LEF2 cells derived from an Eker rat renal tumor [27]. Following low glucose condition (i.e., 2 mM) for 48 hours, a greater proportion of the LEF2 cells died compared with wild-type rat kidney, RK3E, cells (Figure 1A). These results are similar to the findings reported by Inoki et al. [17]. We also tested the effects of 2-deoxyglucose (2-DG) *in vitro *by exposing cells to a low concentration of 2-DG (4 mM) to mimic a clinically achievable concentration under low- (2 mM) and high- (25 mM) glucose concentrations. Figure 1B shows that a reduction of glucose concentration from 25 mM to 2 mM for 6 hours significantly decreased cell viability of both wild-type and mutant cells although the effects were greater in the *Tsc2-/- *cells. The addition of 2-DG accentuated cell death under low-, but not high-, glucose condition such that maximal cell death was achieved in the LEF2 cells when exposed to 2-DG+low-glucose medium. Correspondingly, cellular ATP levels decreased with diminished glucose concentration and 2-DG in an additive manner, and this trend correlated with increased cell death (Figure 1C). Baseline ATP levels were significantly higher in the *Tsc2*-mutant cells, which is consistent with an increased rate of metabolism secondary to mTORC1 activation [19]. The reduction in cellular ATP levels in the LEF2 cells under 'glucose-limiting' conditions was accompanied by an increase in AMPK(Thr172) phosphorylation that was most pronounced under low-glucose+2-DG conditions (Figure 1D). The activation of AMPK was associated with a reduction in p62 expression indicative of autophagy secondary to bioenergetic stress. Wild-type RK3E cells had higher baseline AMPK activity and did not change significantly following 'glucose-restriction'. Together, the *Tsc2-/- *cells were sensitive to glucose restriction and 2-DG in an additive manner.

### "Glucose restriction" on Tsc2-/- tumor growth in vivo

**Figure 2:**
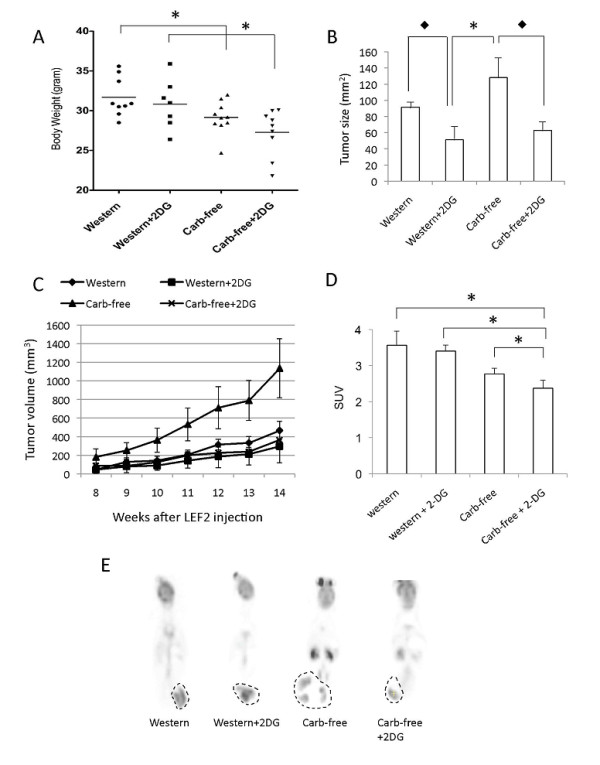
**Effects of 2-DG and diets on LEF2 tumor xenografts**. Eight-week old SCID mice were inoculated with 3 × 10^6 ^LEF2 cells subcutaneously and monitored for 14 weeks under 4 treatment conditions: Western diet, Western diet + 2-DG (0.02%), Carbohydrate-free diet, and Carbohydrate free diet + 2-DG (0.02%). A) Scatter plot of body weights of mice at the end of 14 weeks of treatments. Note that 3 animals in the "Western+2DG" group were sacrificed prior to week 14 due to significant weight loss (i.e., not included in graph). B) Average tumor sizes at week 14 expressed as the product of the length and width. C) Tumor growth over the study period for each of the 4 treatment groups expressed as volume. D) Tumor maximal standard uptake value (SUV) from FDG-PET imaging of 12 mice (3 per group) each with tumor > 5 mm in diameter. E) Examples of FDG-PET coronal images with flank tumors outlined by dotted lines. For graphs, error bars represent SEM. *, p < 0.05; ◆, p = 0.06.

Next, we applied these conditions *in vivo *by treating mice bearing Tsc2-/- tumors with 2-DG and/or a diet without carbohydrates. We predicted that the combination of 2-DG and a "Carb-free" diet would induce a level of energy stress that would suppress tumor growth. In dose-testing experiments, the addition of 0.03% 2-DG by weight to the Western diet led to ~80% of mice with > 20% body weight loss or death over a 4 week period whereas no mouse suffered this degree of weight loss at 0.02% 2-DG. In terms of diets, we used a "Carb-free" diet with 67% of the calories from fat sources, and a 'control' Western diet with 40% fat and 44% carbohydrate as energy (Table 1). Eight-week-old SCID mice were randomly assigned to one of four treatments (n = 10/group): Western diet, Western diet + 2-DG (0.02% wt), "Carb-free" diet, or "Carb-free" diet + 2-DG (0.02% wt). After 4 days of acclimatization to the diets, each mouse was inoculated subcutaneously with 3 × 10^6 ^LEF2 cells in the dorsal flank region. Over 14 weeks of observation, one animal from each group, except the "Carb-free" diet group, died during this period without evidence of excessive tumor burden or body weight change (i.e., random deaths). Three animals in the Western+ 2DG group showed significant weight loss over the experimental period requiring early termination between 10-11 weeks. The remaining 6 mice in the Western+2DG group had body weights that were similar to the other groups at the end of 14 weeks (Figure 2A). The "carb-free" diet also reduced body weight compared to the Western diet (Figure 2A).

**Table 1:** Composition of the Western and "Carb-free" diets*.

	Western	"Carb-free"
***Energy ***(kcal/g)		

Protein	16.1%	33.2%

Fat	39.7%	66.8%

Carbohydrates	44.2%	0%

***Ingredients ***(%)		

Corn starch	31.2	0

Casein	19.7	48.1

Dextrin	10.4	0

Sucrose	7.9	0

Vegetable Shortening	6.1	11.8

Milk fat	6.1	11.8

Lard	6.1	11.8

AIN93G Mineral mix/fiber	4.0	4.7

Inulin	2.5	2.9

Powdered Cellulose	2.5	2.9

Soybean Oil	1.3	2.5

AIN93 Vitamin mix/fiber	1.2	1.3

Corn Oil	0.5	1.1

L-Cystine	0.3	0.7

Choline bitartrate	0.2	0.2

Cholesterol	0.1	0.2

*Source: www.testdiet.com

Western: AIN-93G Western diet (5TJN), "Carb-free": AIN-93G Ketogenic diet (5TJR))

At time of sacrifice, tumor sizes were significantly different among the 4 groups (ANOVA, p < 0.05) (Figure 2B). In pair-wise comparisons, tumor sizes of the Western+2DG and the 'Carb'-free groups were significantly different (p < 0.05) while the 2-DG-treated tumors showed strong trends towards smaller size compared to each of the respective diets (p = 0.06) (Figure 2B). Tumor size at the end of 14 weeks and growth rate of tumors were highest in the "Carb-free" group (Figure 2B, C). Analyses of variance did not reveal a significant 'interaction' between the two treatments (i.e., 2-DG and diet). Thus, our initial hypothesis of superior tumor control through the combination of glycolytic inhibition and carbohydrate restriction is not supported by the data. Instead, we found that 2-DG suppressed tumor growth regardless of diet composition. Unexpectedly, carbohydrate restriction led to a promotion in tumor size despite a loss in overall body weight.

### Influence of diet and 2-DG on tumor FDG uptake

Recognizing that the prescribed treatments may impact tumor glucose metabolism, we performed FDG-PET on a subset of the 12 mice (3 per group) bearing tumors of sufficient size (i.e., > 5 mm) for detection with PET imaging. Following an overnight fast, mice were given 200 μCi of ^18^FDG intravenously and scanned after a 45-minute uptake period. FDG activity was normalized to body weight and time of FDG injection and expressed as standard uptake value (SUV). Figure 2E illustrates representative PET images from each of the 4 treatments showing significant heterogeneity. We used the maximal SUV value within each tumor to indicate the metabolic activity of the lesions. The mean SUV_max _of the LEF2 tumors were significantly different among the 4 groups with the highest values found in the Western diet group and the lowest in the Carb-free + 2-DG group (Figure 2D). While carbohydrate restriction and 2-DG reduced FDG uptake, SUV_max _did not correlate with tumor size (compare Figures 2B with2D). The paradoxical increase in tumor size with reduced tumor SUV_max _in the "Carb-free" group suggests the possibility of alternative energy sources besides glucose that were utilized by the *Tsc2-/- *tumor cells.

### Ketone bodies support Tsc2-/- cell growth in vitro

**Figure 3:**
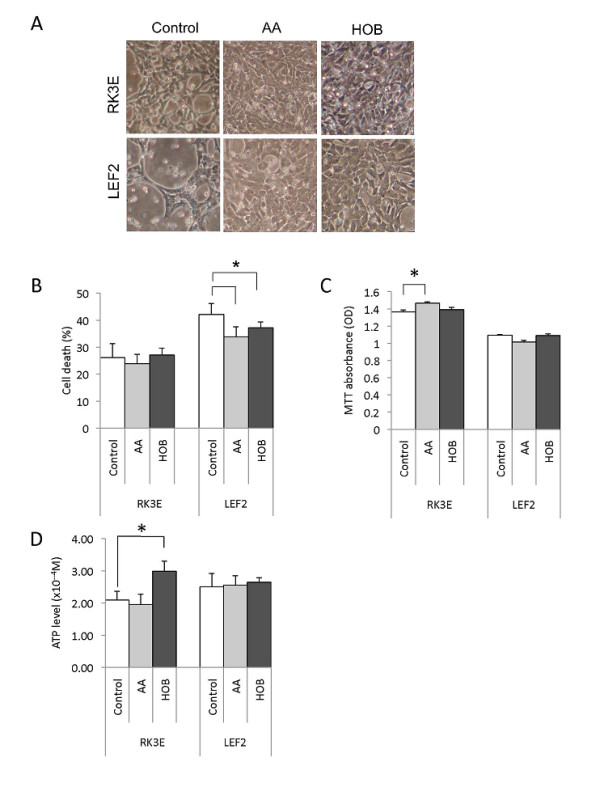
**Influence of ketone bodies on LEF2 cells**. A) Appearance of cells under phase contrast microscopy following 2 days of exposure to acetoacetate (AA, 1 mg/ml), β-hydroxybutyrate (HOB, 2.5 mg/ml), or vehicle control under low-glucose (2 mM) condition. Magnification 10×. Effects of ketone bodies on B) cell death as determined by trypan blue exclusion assay, C) proliferation (MTT assay) and D) ATP levels. For graphs, error bars represent SEM. *, p < 0.05.

Diets devoid of carbohydrate may lead to an imbalance between β-oxidation of fatty acids and carbohydrate metabolism resulting in ketogenesis [26]. To examine the effects of ketone bodies on LEF2 cells, we supplemented the low-glucose (2 mM) culture media with either acetoacetate (AA, 1 mg/ml) or β-hydroxybutyrate (HOB, 2.5 mg/ml). After 1 day in culture, both LEF2 and RK3E cells showed greater confluence compared to cells grown in 2 mM glucose alone (Figure 3A). Using trypan blue exclusion assay, the proportion of non-viable LEF2 cells was moderately reduced in the presence of ketone bodies (Figure 3B) whereas MTT absorbance remained relatively constant (Figure 3C). β-hydroxybutyrate supplementation was accompanied by an increase in ATP levels in the wild-type cells but had little effect in the LEF2 cells (Figure 3D).

**Figure 4:**
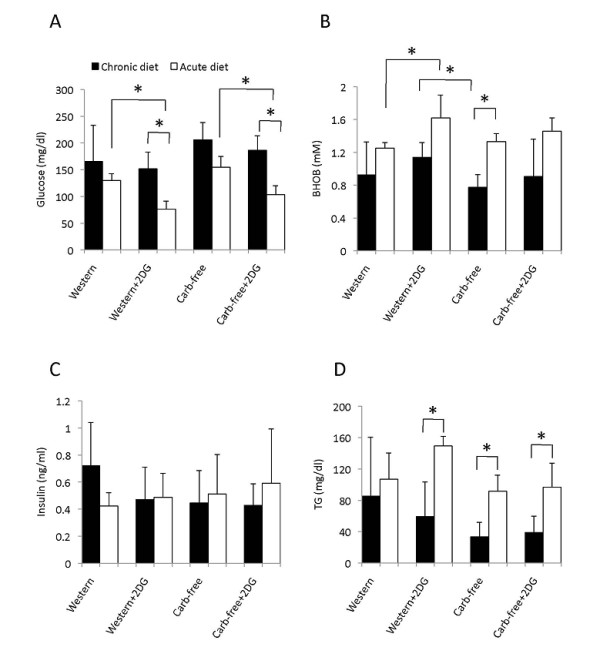
**Systemic metabolic responses to diets and 2-DG**. Sera obtained at sacrifice in fasted animals was analyzed for A) glucose, B) β-hydroxybutyrate (BHOB), C) insulin and D) triglyceride (TG). Chronic diet (black bars) indicates those that were treated for 14 weeks. Acute diet (open bars) indicates treatments for 4 days. Error bars represent SEM. *, p < 0.05.

To assess the relevance of these findings *in vivo*, we measured serum β-hydroxybutyrate levels after 14 weeks of treatments. Contrary to expectation, the "Carb-free" diet did not induce ketogenesis but rather, had the lowest average β-hydroxybutyrate and highest glucose levels (Figure 4A, B) while serum triglyceride trended lower (Figure 4D) and insulin level remained unchanged (Figure 4C). On the other hand, those receiving Western diet + 2-DG had the highest β-hydroxybutyrate and lowest glucose levels. To confirm that the lack of ketonemia in the "Carb-free" fed animals was not due to systemic adaptation following chronic exposure, we conducted a short-term experiment (e.g., 4 days) using the same treatment protocol. The 'acute' changes in serum glucose and β-hydroxybutyrate paralleled the trends found in the long-term experiment (Figure 4). Again, the "Western+2-DG" diet induced the largest metabolic changes in terms of hypoglycemia and ketonemia. These observations are consistent with the significant weight loss associated with "Western+2-DG" treatment. Our data also indicate that neither acute nor chronic ingestion of the carbohydrate-free diet was sufficient to induce ketogenesis in mice. Although the *Tsc2*-null cells can adapt to using ketone bodies *in vitro *to support cell survival, the accelerated tumor growth in the "Carb-free" group was not associated with ketonemia.

### Role of fatty acids in the "Carb-free"-fed tumors

**Figure 5:**
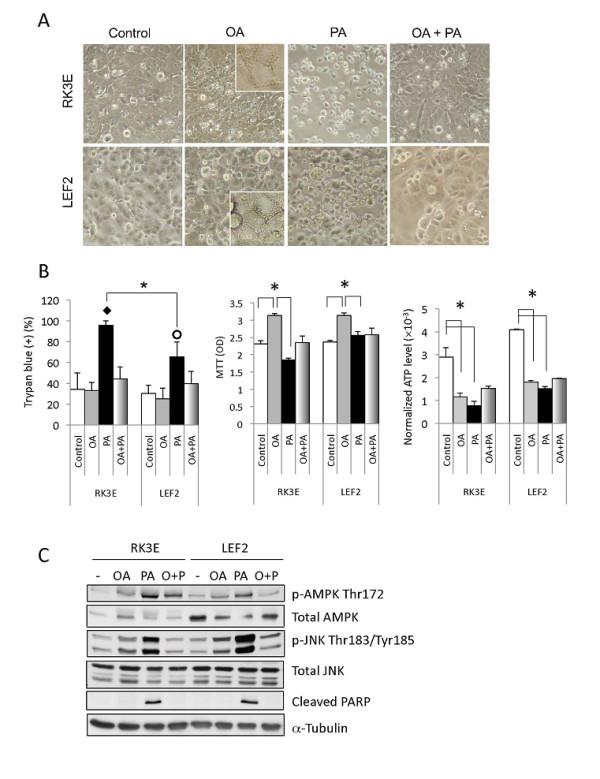
**Effects of fatty acids on LEF2 cells**. A) Appearance of cells 24 hrs following exposure to oleic acid (OA, 1 mM), palmitic acid (PA, 1 mM), both (OA+PA, 3 mM at 2:1 ratio) or vehicle control. Magnification 10×. Inserts represent higher magnification views showing cytoplasmic vesicles. B) Assessment of cell viability (Trypan blue exclusion), proliferation (MTT), and ATP levels following 6 hours of fatty acids exposure. C) Western blot analyses of cell lysates from each of the four treatments blotted for the indicated proteins. α-tubulin serves as a loading control. For graphs, error bars represent SEM. *, p < 0.05; ◆ indicates p < 0.01 compared to all other groups; O indicates p < 0.05 compared to all other groups.

Since the "Carb-free" diet is composed primarily of fat (i.e., 67%), of which 40% is saturated fatty acids, its absorption from the intestine provides a direct source of fatty acids that can serve as energy substrates. To examine the effects of fatty acids on LEF2 cells, we incubated cells with monounsaturated oleic acid and/or saturated palmitic acid in the presence of 25 mM glucose for 24 hours. Exposure to oleic acid led to the accumulation of cytoplasmic vesicles and cell proliferation (Figure 5A, B). On the other hand, palmitic acid induced cell death in 63% and 94% of LEF2 and RK3E cells, respectively and reduced cell proliferation of RK3E but not LEF2 cells (Figure 5A, B). The addition of oleic acid to cultures with palmitic acid significantly improved cell viability. Cellular ATP levels decreased in the presence of fatty acids, but more so with palmitic acid (Figure 5B). Corresponding to the reduced energy state, AMPK and JNK phosphorylation were induced most prominently by palmitic acid, and this was accompanied by the expression of cleaved PARP (Figure 5C). Therefore, monounsaturated oleic acid and saturated palmitic acid have contrasting effects on cell viability and proliferation. These findings correlate with our *in vivo *observation that tumors in the "Carb-free" group showed increased areas of necrosis and liquefaction (see below).

### 2-DG reduces tumor proliferation

**Figure 6:**
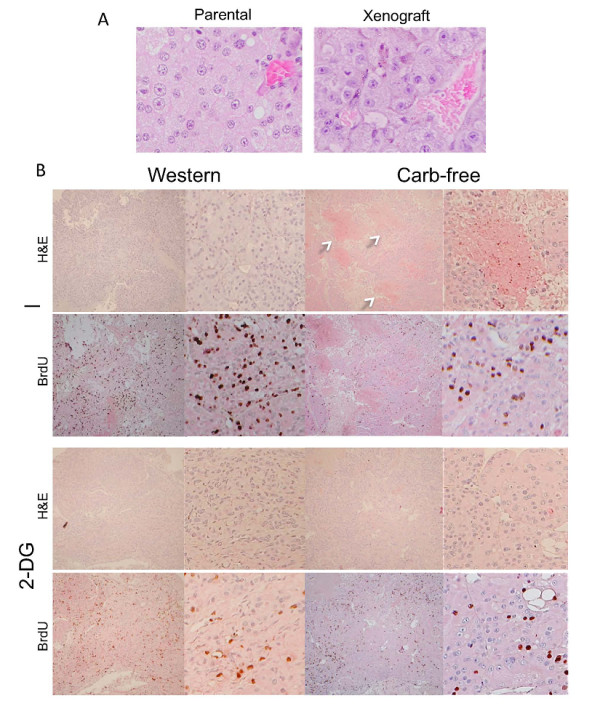
**Morphologic appearances of LEF2 tumor xenografts**. A) Comparison of parental renal cell tumor from an Eker rat and a LEF2 xenograft. Shown are representative sections of H&E-stained, formalin-fixed tumors. Magnification 400×. B) Representative microscopic appearances of the LEF2 xenografts at time of sacrifice from each treatment group. Shown are low- (left) and high- (right) magnification views of the H&E and BrdU staining for each of the 4 treatment groups. Note the hemorrhagic and necrotic zones (arrows) in the Carb-free sample.

Histologically, the LEF2 tumor xenografts in the 4 treatment groups shared features of the parent tumor with large, eosinophilic cells and prominent nucleoli accompanied by abundant tumor vessels (Figure 6A). However, the larger tumors in the "Carb-free" group had significantly more necrotic areas that correlated with a soft texture on gross examination (Figure 6B). On sectioning, these tumors contained liquefied, hemorrhagic materials that correlated with the 'patchy' FDG-uptake on PET (see Figure 2E).

**Figure 7:**
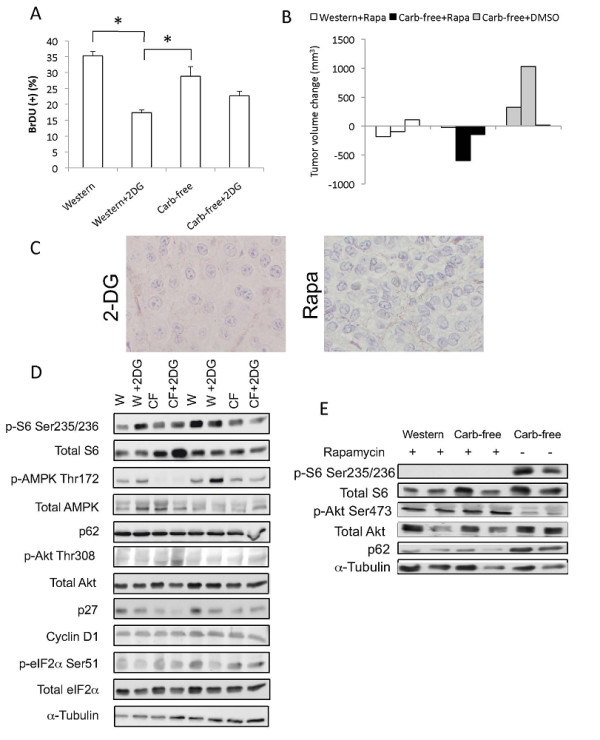
**Effects of 'glucose deprivation' on tumor proliferation, response to rapamycin and signaling**. A) Tumor proliferation rates based on BrdU incorporation. BrdU (50 μg/g) was injected intraperitoneally once daily for two days before sacrifice. Histologic sections were stained with anti-BrdU antibodies. For each sample, > 1,000 tumor cells were counted to determine the fraction of tumor cells with positive nuclear BrdU staining. *, p < 0.05. B) Response of the LEF2 tumors to rapamycin treatment (Rapa, 1 mg/kg - 5 days a week for 2 weeks) based on change in tumor volume. Three mice per group. C) Representative photomicrographs of LEF2 xenografts following 2-DG and rapamycin (Rapa) treatment. Magnification 400×. Note differences in cell size. D) Immunoblot of representative tumor lysates from the 4 treatment groups (2 each) showing the expression of the indicated proteins. W, Western diet; CF, carbohydrate-free diet. E) Western blot of tumor lysates from (B) showing effects of rapamycin on mTORC1 (p-S6), Akt (p-Akt), and autophagic (p62) pathways.

We determined the rate of cell proliferation by counting the number of cells with nuclear BrdU staining following *in vivo *labeling (Figure 6B). With over 1,000 cells counted for each group, the proportion of BrdU(+) cells was significantly reduced with 2-DG treatment (Figure 7A). Tumors from the Western diet-fed mice had the highest proliferative rate but this was not significantly different from those fed "Carb-free" diet. Since the "Carb-free" group with the largest tumors did not have the highest proliferative rates, other processes beside cell proliferation may be influencing tumor size. Neither cell size nor tumor CD34+ structures were significantly different between the treatment groups (data not shown). Instead, tumors in "Carb-free" diet group displayed necrosis and liquefaction probably on the basis of saturated fatty acids. This may have contributed to the increase in overall tumor size. Nonetheless, "Carb-free" tumors contained a significant amount of viable cells since rapamycin effectively reduced the tumor sizes in the "Carb-free"-diet group to the same extent as the "Western"-diet group (Figure 7B). Histologically, rapamycin-treated tumors showed significant reduction in cell size whereas 2-DG primarily decreased proliferation, thus highlighting differences in anti-tumoral mechanisms (Figure 7C).

Finally, we compared the relative activities of the Akt/mTOR and AMPK pathways in LEF2 tumors following the four treatments. Tumor lysates uniformly showed high levels of phospho-S6 indicative of mTORC1 activation and low levels of phospho-Akt secondary to feedback inhibition [7] with no consistent diet/treatment-related effects (Figure 7D). Thus, the anti-tumoral effects of 2-DG was not secondary to mTORC1 inhibition. AMPK phosphorylation was most pronounced following exposure to the "Western diet+2-DG" treatment in keeping with its associated metabolic stress (e.g., ketonemia) and our *in vitro *findings (see Figure 1D, 4). However, this did not correlate with changes in the levels of phospho-S6, p27, or cyclin D1. Further, there was no consistent alterations in p62 or phospho-eIF2α expression in tumor lysates to suggest induction of autophagy or ER stress, respectively. In contrast, rapamycin abolished S6 phosphorylation, increased Akt phosphorylation and reduced p62 expression to an equal extent in tumors from the "Western" and "Carb-free"-diet groups (Figure 7E); this is consistent with the known effects of rapamycin in promoting autophagy [28].

## Discussion

We investigated two approaches aimed at restricting glucose metabolism in the treatment of TSC-related tumors. The rationale of our study is based on the observations that the loss of TSC1/TSC2 leads to an energy imbalance caused by mTORC1 hyperactivity that increases energy demand stemming from macromolecular synthesis and reduces energy supply as a result of impaired insulin-stimulated glucose uptake [19,20]. Consequently, cells deficient in TSC1 or TSC2 are prone to undergo apoptosis upon glucose withdrawal or 2-DG treatment [17]. The combination of low glucose concentration and 2-DG was more effective in limiting ATP, activating AMPK and inducing cell death *in vitro *(Figure 1). When applied to an *in vivo *model of *Tsc2*-null tumor, 2-DG suppressed tumor growth by reducing cell proliferation whereas a diet free of carbohydrate resulted in larger tumors with increased zones of liquefaction. We did not detect an 'additive' effect when the two treatments were given together. These findings indicate that the *Tsc2*-related tumors are sensitive to glycolytic inhibition *in vivo*, but the lack of tumor suppression following dietary restriction of carbohydrate could be a reflection of its inability to induce significant hypoglycemia. On the other hand, the increased necrosis in the "Carb-free" fed tumors was not due to an effect of the diet on tumor vasculature, ER stress or autophagy since the tumor expression of CD34, phospho-eIF2α(Ser51) and p62 were not significantly different among the 4 groups.

The ketogenic diet has been investigated for over two decades as a treatment for malignant brain tumors based on the notion that tumor metabolism favors aerobic glycolysis (i.e., Warburg effect) whereas tissues of critical organs such as the brain can utilize ketone bodies efficiently [29]. In a short-term study, Marsh et al. reported a synergistic effect of 2-DG and a carbohydrate-free diet on inhibiting astrocytoma growth in a mouse model. They attributed the benefit of the diet to energy restriction resulting in ketogenesis and significant weight loss [30]. When diet was given without restriction, its composition did not appear to significantly impact tumor growth *in vivo *[31]. In our study, mice receiving the "Carb-free" diet weighed less than the Western diet group, but the difference was only ~10%, and the β-hydroxybutyrate levels did not rise significantly. Thus, unrestricted "Carb-free" diet per se did not create a state of metabolic "stress" in mice. In addition to the lack of caloric restriction, our "Carb-free" diet contained high protein content that can serve as an energy source via gluconeogenesis. Mavropoulos et al. also failed to show a significant anti-tumoral effect of the no-carbohydrate ketogenic diet compared with a Western diet in a prostate cancer xenograft model despite a decrease in serum insulin, IGF-1 and phospho-Akt levels [32]. Interestingly, the ketogenic diet used in controlling seizures has been associated with disease progression in 3 of 5 human TSC patients [33]. Together, these findings should raise awareness for more vigilant monitoring of TSC-related pathology in patients receiving the ketogenic diet for seizure management. At present, the effects of caloric restriction in TSC-related tumors are not known, but we surmise the possibility that a caloric-restricted diet with reduced glucose (exogenous or endogenous) availability may synergize with 2-DG to limit tumor growth.

Inhibition of glycolysis by 2-DG is currently being tested in clinical trials. 2-DG causes a depletion of cellular ATP that leads to the activation of AMPK. In combination with a Western diet, 2-DG treatment had the most severe metabolic stress in terms of hypoglycemia and ketogenesis. Consequently, we encountered a greater degree of toxicity with 3 of 10 mice experiencing significant weight loss before the completion of the experiment. This metabolic consequence was not observed in the mice receiving 2-DG while fed a "Carb-free" diet. Significant weight loss occurs when glycolysis is inhibited in which carbohydrate constitutes a significant source of energy as in the Western diet. When glucose becomes limiting, the body mobilizes fat stores for energy (i.e., lipolysis) resulting in loss of subcutaneous fat and weight, which is what we observed in a subset of the Western+2DG group. In contrast, the "Carb-free" diet provides abundant fat and protein as energy substrates, thus limiting the severity of weight loss. With respect to the Western diet+2DG treatment, AMPK(Thr172) phosphorylation was increased under *in vitro *and *in vivo *conditions, but we did not detect significant suppression of mTORC1 activity or changes in p27 and cyclin D1 expression. Therefore, the mechanism of 2-DG inhibition on cell proliferation *in vivo *remains undefined. Compared to rapamycin-treated samples, two distinct patterns of response emerged with 2-DG slowing proliferation and rapamycin reducing cell size. However, it is unlikely that the combination of 2-DG and rapamycin would be beneficial since the effects of mTORC1 inhibition would neutralize the glucose-dependence of the *Tsc2*-null cells. Indeed, Inoki et al. have shown that rapamycin and re-expression of Tsc2 in LEF2 cells rescued them from apoptosis under low-glucose condition [17].

Besides glucose, we found that ketone bodies can be utilized by cells *in vitro *to promote survival and growth although these effects were mild (Figure 3). In contrast, Fine et al. reported that ketone bodies inhibit cell growth by reducing ATP concentration in tumor cells that over-express UCP2 [34]. We also showed that cell proliferation can be augmented by oleic acids in culture despite a reduction in cellular ATP, but saturated palmitic acid was toxic to cells. These findings are in keeping with those reported by Ricchi et al. in hepatocytes [35]. Considered together, the *Tsc2*- tumor cells exhibit metabolic plasticity beyond their dependence on glycolysis. This may explain the lack of correlation between FDG uptake and tumor response. Further, glutamine has also been identified as an important nutrient in supporting cellular bioenergetics of the *Tsc2*-null cells [18]. In future studies, targeting multiple metabolic pathways may be considered to maximize tumor control while minimizing toxicity.

## Conclusions

Our study provides the first *in vivo *evidence demonstrating anti-tumoral effects of glycolytic inhibition in TSC2-related tumors. The predominant effect of 2-DG was to inhibit cell proliferation in contrast to the effect of rapamycin on cell size. A diet free of carbohydrate without caloric restriction was not effective in controlling TSC2-tumor growth, but changed the consistency of the tumors by inducing necrosis.

## Methods

### Cells and reagents

LEF2 cells were derived from an Eker rat renal tumor (*Tsc2-/-*), and the wild-type rat kidney-derived cells, RK3E, were purchased from ATCC. Both were cultured in DMEM/F12 medium containing 10% FBS fetal bovine serum (FBS), 100 units/ml penicillin G and 100 μg/ml streptomycin sulfate at 37°C in a humidified 5% CO_2 _incubator. Antibodies were purchased from the following sources: anti-BrdU (Dako), anti-CD34 (Cedarlane, Burlington, NC), anti-p62 and anti-α-tubulin (Sigma, St. Louis, MO). All remaining antibodies (anti-AMPK, anti-phospho-AMPK(Thr172), anti-S6, anti-phospho-S6, anti-p27, anti-cyclin D, anti-eIF2α, anti-Akt, anti-phospho-Akt, anti-cleaved PARP, and anti-cleaved-caspase 3) were from Cell Signaling (Danvers, MA). 2-deoxyglucose, oleic and palmitic acids were purchased from Sigma (St. Louis, MO). Rapamycin were obtained from EMD Biosciences (San Diego, CA).

### Xenograft model of *Tsc2-*null tumor

Young (5-6 weeks), male, NOD.CB17-prkdcscid/J mice were purchased from JAX and fed regular chow for 2-3 weeks. At 8 weeks of age, animals were randomly allocated to 4 treatment groups: Western diet, Western+0.02% 2-DG, 'Carb-free' diet, 'Carb-free'+0.02% 2-DG. The diets with and without 2-DG were prepared and purchased from Animal Specialties (Hubbard, OR). After 4 days on treatment, 3 × 10^6 ^LEF2 cells were injected subcutaneously in the flank region of the mice. Animals were monitored for tumor growth and general health for 14 weeks. After an overnight fast, mice were sacrificed, tumors and sera were collected for analyses. For a subset of 12 animals, FDG-PET was performed (see below) and BrdU (50 μg/g) was injected intra-peritoneally once daily for two days before sacrifice. All work related to animals was in accordance with a protocol approved by the Institutional Animal Care Committee, University of Washington, Seattle.

### FDG-PET imaging

Following an overnight fast, mice were anesthetized using isoflurane and given a retro-orbital injection of FDG (200 μCi). Mice were kept warm and anesthetized during a 45-minute uptake period. Following this, mice were imaged for 20 minutes on a Siemens Inveon Dedicated PET system at the micro-PET imaging facility (University of Washington). A transmission scan was taken of each mouse after the emission study. Images were reconstructed using the manufacturer supplied 3D OSEM MAP image reconstruction. Data were collected with both random, scatter and attenuation correction. A Beta smoothing parameter of 0.1 was used. The maximum uptake value for each tumor was captured from the reconstructed images by stepping through the tumor and measuring the maximum activity (nCi/cc) in all hotpots and was normalized to body weight and time of FDG injection (to account for decay) in order to calculate the standard uptake value (SUV).

### Trypan Blue Exclusion Assay

Quantification of viable cells was performed using the trypan blue exclusion method. In brief, cells were harvested and collected following trypsin detachment and centrifugation. Cells were rinsed with PBS and then resuspended in 1 ml of PBS. A 10-μl aliquot of cell suspension was incubated with 10 μl 0.4% trypan blue stain (Invitrogen/Gibco) solution for 5 minutes at room temperature. Viable and nonviable cells based on absence and presence of intracellular dye, respectively, were counted by hemacytometer. Results represent the number of nonviable cells divided by the total number of cells counted and are expressed as percentages.

### Flow Cytometric Analyses

Cell apoptosis was measured by flow cytometry using the Annexin V kit (AbD Serotec, Raleigh, NC). In brief, RK3E and LEF2 cells were detached following treatment with 0.05% trypsin-EDTA for 10 minutes at 37°C, washed with PBS, pelleted by centrifugation, resuspended with pre-diluted binding buffer, and stained with Annexin V-FITC for 10 minutes in the dark at room temperature. Cells were washed and resuspended in binding buffer with Propidium lodide solution and analyzed by flow cytometry.

### ATP level measurement

Cells were seeded on plates with 10% FBS with DMEM/F12 medium for overnight, then washed with PBS and replaced with 10% dialyzed FBS DMEM with 2 mM glucose, 1 mM HEPES. After 1 day in culture medium, cells were treated with 4 mM 2-Deoxyglucose, 1 mg/ml acetoacetate, or 2.5 mg/ml β-hydroxybutyrate for 6 hours. ATP levels were measured using a Luminescence ATP detection assay system (PerkinElmer, MA). Briefly, cells were lysed for 5 minutes with supplied lysis buffer in an orbital shaker at 700 rpm, then ATP detection substrate solution was added to the wells and agitated for 5 minutes. Plates were left in the dark for 10 minutes and luminescence was measured using a Molecular Devices Spectra Max M2 (Sunnyvale CA).

For fatty acid experiments, oleic acid-BSA and palmitic acid-BSA solutions were pre-made (2 moles of FFA to 1 mole BSA). Cells were washed with PBS and cultured in 10% charcoal striped FBS phenol-free DMEM with 1 mM Oleic acid-BSA, 1 mM palmitic acid-BSA, or 2 to 1 molar of oleic acid/palmitic acid (3 mM) for 24 hours.

### MTT assay

Cells were grown on 24-well plates in 10% FBS DMEM/F12, treated with ketone bodies (1 mg/ml acetoacetate or 2.5 mg/ml β-hydroxybutyrate for 24 hours) or FFA (1 mM oleic acid, 1 mM palmitic acid or 3 mM oleic acid + palmitic acid for 24 hours) followed by MTT proliferation assay. Briefly, 100 μl of 5 mg/ml MTT was added to each well and incubated for 3.5 hours. 750 μl of MTT solvent (4 mM HCl, 0.1% Nondet P-40 in isopropanol) was added to each well and agitated for 15 minutes. Optical density was read at 600 nm using a Packard Spectracount microplate reader (PerkinElmer, Inc., Waltham, MA).

### Serum biochemistry

Blood was extracted via cardiac puncture immediately after sacrifice. Blood was spun for 15 minutes at 3000 rpm at 4°C. Plasma was analyzed for glucose, insulin, β-hydroxybutyrate, and triglycerides. Plasma glucose levels were measured colorimetrically using the glucose oxidase reagent (Pointe Scientific, Canton, MI). Plasma insulin levels were measured using the Linco ELISA (Millipore, Billerica, MA). β-hydroxybutyrate in plasma was measured using the KetoSite β-hydroxybutyrate dehydrogenase assay according to the manufacturer's instructions (Stanbio Laboratory, Boerne, TX). Plasma triglycerides were quantified via the Wako L-Type TG M colorimetric assay (Wako Diagnostics, Richmond, VA).

### Histology and immunohistochemistry

Portions of tumor were fixed in either neutral buffered formalin for routine histology and immunohistochemistry (IHC) or in methacarn (60% methanol, 30% chloroform, and 10% acetic acid) for bromo-deoxyuridine (BrdU) labeling. After fixation, tissue was embedded in paraffin. For routine histological analysis, 5-μm sections were cut from paraffin-embedded blocks and sections were deparaffinized, rehydrated, and washed before staining with hematoxylin QS and eosin (Vector Laboratories, Burlingame, CA) and mounted with Permount (Fischer Scientific, Santa Clara, CA). For CD34 staining, sections were deparaffinized, rehydrated, and washed with phosphate-buffered saline. After antigen retrieval in 0.1 M sodium citrate (pH 6.0) and quenching of endogenous peroxidase activity with 3% H_2_O_2_, samples were blocked with 5% normal horse serum (NHS) before incubation with primary antibodies overnight at 4°C. Negative controls were treated with 5% NHS without primary antibodies. Staining was detected using the Elite Vectastain ABC kit (Vector laboratories, Burlingame, CA) according to the manufacturers instructions. BrdU IHC was performed as described above with the following modifications: endogenous peroxidase activity was quenched with 0.3% H_2_O_2_/Methanol for 10 minutes and antigen retrieval consisted of trypsin digestion (1 mg/ml trypsin for 10 min at room temperature), followed by incubation in 2.5 M HCl for 10 min at 37°C. Mouse anti-BrdU diluted 1:40 was used as the primary antibody (Dako, Carpinteria, CA, USA).

### Immunoblot analyses

Cells and tumor tissues were homogenized in ice-cold radioummunoprecipitation (RIPA) buffer (1% Nonidet P-40, 1% sodium deoxycholate, 0.1% SDS, 0.15 M NaCl, 10 mM Tris (pH 7.2), 0.025 M β-glycophosphate (pH 7.2), 2 mM EDTA, and 50 mM sodium fluoride) with protease and kinase inhibitors (0.05 mM AEBSF, 10 μg/ml aprotinin, 10 μg/ml pepstatin, 1 mM orthovanadate, 10 μg/ml leupeptin, 1 mM microcystin LR). The protein concentration was measured using the BCA Protein Assay (Pierce, Rockford, IL). Equal amounts of protein were separated by SDS-PAGE, transferred to Immobilin-P membranes (Millipore, Bedford, MA) and blotted with antibodies according to manufacturer recommendations.

### Statistical analyses

Comparisons between two groups were analyzed using the Student t-test. For multi-group comparisons, analysis of variance (ANOVA) was used.

## List of abbreviations

TSC: tuberous sclerosis complex; mTORC1: mammalian target of rapamycin complex 1; 2-DG: 2-deoxyglucose; AMPK: adenosine monophosphate-activated protein kinase; PET: positron emission tomography; SUV: standard uptake value.

## Competing interests

The authors declare that they have no competing interests.

## Authors' contributions

XJ performed the in vitro and in vivo studies and their analyses. HLK assisted in the in vivo experiments and performed histologic analyses. RSY conceived of the study, oversaw the design and execution of the experiments, helped with the analyses and manuscript preparation. All authors read and approved the final manuscript.
